# The Tomato Nucleotide-binding Leucine-rich Repeat Immune Receptor I-2 Couples DNA-binding to Nucleotide-binding Domain Nucleotide Exchange*

**DOI:** 10.1074/jbc.M115.698589

**Published:** 2015-11-24

**Authors:** Stepan Fenyk, Christopher H. Dixon, William H. Gittens, Philip D. Townsend, Gary J. Sharples, Lars-Olof Pålsson, Frank L. W. Takken, Martin J. Cann

**Affiliations:** From the ‡ School of Biological and Biomedical Sciences,; the § Biophysical Sciences Institute, and; the ¶ Department of Chemistry, Durham University, South Road, Durham DH1 3LE, United Kingdom and; the ‖ Molecular Plant Pathology, Swammerdam Institute for Life Sciences, University of Amsterdam, 1098 XH, Amsterdam, The Netherlands

**Keywords:** ATPases associated with diverse cellular activities (AAA), cellular immune response, DNA binding protein, Nod-like receptor (NLR), nucleotide, plant biochemistry

## Abstract

Plant nucleotide-binding leucine-rich repeat (NLR) proteins enable plants to recognize and respond to pathogen attack. Previously, we demonstrated that the Rx1 NLR of potato is able to bind and bend DNA *in vitro*. DNA binding *in situ* requires its genuine activation following pathogen perception. However, it is unknown whether other NLR proteins are also able to bind DNA. Nor is it known how DNA binding relates to the ATPase activity intrinsic to NLR switch function required to immune activation. Here we investigate these issues using a recombinant protein corresponding to the N-terminal coiled-coil and nucleotide-binding domain regions of the I-2 NLR of tomato. Wild type I-2 protein bound nucleic acids with a preference of ssDNA ≈ dsDNA > ssRNA, which is distinct from Rx1. I-2 induced bending and melting of DNA. Notably, ATP enhanced DNA binding relative to ADP in the wild type protein, the null P-loop mutant K207R, and the autoactive mutant S233F. DNA binding was found to activate the intrinsic ATPase activity of I-2. Because DNA binding by I-2 was decreased in the presence of ADP when compared with ATP, a cyclic mechanism emerges; activated ATP-associated I-2 binds to DNA, which enhances ATP hydrolysis, releasing ADP-bound I-2 from the DNA. Thus DNA binding is a general property of at least a subset of NLR proteins, and NLR activation is directly linked to its activity at DNA.

## Introduction

Plants rely on an innate immune system to ward off pathogens (1–4). Pathogen perception and recognition specificity is typically controlled by NLR^3^ type immune receptors that are capable of perceiving non-self and modified self molecules inside the host cell. NLRs typically detect strain-specific pathogen effectors or the effect these virulence factors exert on host proteins (3, 5, 6). NLR proteins are members of the STAND (signal transduction ATPases with numerous domains) P-loop ATPases of the AAA+ (ATPases associated with diverse cellular activities) superfamily whose multidomain structure allows them to function CCsimultaneously as sensor, switch, and response factor (7, 8).

Plant NLRs are named after their central nucleotide-binding (NB) and C-terminal leucine-rich repeat (LRR) domains. The N terminus of a plant NLR is extremely divergent and typically encodes either a coiled-coil (CC) or Toll interleukin receptor domain (3). In addition to these core domains, other domains can be present such as a WRKY, Squamosa promoter binding protein-like heavy metal-associated or Ratx1 (related to ATX1) domains that, in conjunction with the LRR, are proposed to aid in effector perception (9–11). The LRR is hypothesized to maintain the NLR protein in a signaling competent yet autoinhibited state (12). The central NB domain, also referred to as the NB-ARC domain because of its homology with APAF1, CED4, and R proteins, is predicted to function as a nucleotide-operated molecular switch controlling the signaling activity of the protein (8, 13, 14). Biochemical analysis of tomato I-2 and Mi-1, flax M and L6, and barley MLA27 revealed that in the autoinhibited “off” state, the NB-ARC domain is ADP bound (15–17). Upon pathogen recognition, ADP is exchanged for ATP permitting the NB-ARC domain to adopt an activated and structurally distinct “on” state. Hydrolysis of bound ATP into ADP enables the off state to be re-established. There are several lines of supporting evidence for this model. For example, tomato I-2 mutants defective in ATP hydrolysis *in vitro* are autoactive *in vivo* (16). Further, an autoactive flax M mutant preferentially co-purifies with ATP (17). Interestingly, the NLR NB-ARC domain is not necessarily a strict ATPase. The NB subdomain of rice Os2g_25900, NB-ARC domains of maize PSiP (pollen-signaling protein), and *Arabidopsis* Rpm1 possess a nucleotide phosphatase activity (18). The described phosphatase sequentially removes terminal phosphates from the nucleotide to form the nucleoside in a reaction that remains compatible with the switch model.

How activated plant NLRs trigger immune signaling is a crucial and largely unanswered question. Activation of animal NLRs typically induces multimerization, resulting in the formation of a cytoplasmic signaling scaffold on which partners are activated because of their induced proximity (19). Unlike animal NLRs, no conserved protein binding partners for plant NLRs have been identified to date that could play an analogous role in downstream signaling. The identified NLR interactors are mostly involved in processes such as NLR protein maturation and folding, nucleocytoplasmic shuttling, or effector perception (9, 12, 20). Whereas NLR proteins locate at various subcellular localizations determined by the necessity to intercept effector action at distinct locations, the subcellular localization from which primary immune signaling is activated is not unambiguously resolved. Although some NLRs are confined to a specific subcellular compartment, like the plasma membrane localization of Rpm1 and the nuclear localization of RRS1-R (21, 22), other NLRs have a more dynamic distribution. For instance tobacco N, barley MLA1 and Mla10, *Arabidopsis* RPS4 and SNC1, and potato Rx1 show a nuclear-cytoplasmic distribution (22–29). The functional importance of nuclear localization is emphasized by genetic screens that reveal genes encoding components of the nuclear pore complex to be required for NLR-mediated resistance (30, 31). Furthermore, redistribution of MLA10, N, RPS4, and SNC1 from the nucleus to the cytoplasm compromises their immune signaling (24, 25, 27, 31), suggesting that their signaling target resides in the nucleus. Together with the recent notion that many NLRs work in pairs, such as RRS-1/RPS4 and RGA4/RGA5 or require “downstream” or “helper” NLRs such as ADR1, NRG1, or NRC members, this suggests the interesting hypothesis that, even for NLRs not located themselves in the nucleus, the conserved signaling target for NLR signaling might be a nuclear component (32–36).

In line with this hypothesis, we recently identified DNA as a molecular target for an activated NLR. We demonstrated that the potato Rx1 NLR possesses an intrinsic DNA binding and melting activity *in vitro* (37). In addition, Rx1 was observed to bend and locally melt dsDNA. DNA binding is mediated by its NB-ARC domain in a manner similar to that of the structurally homologous origin of replication binding proteins. An Rx1-DNA interaction in plants was only found upon activation of immune signaling by the Rx-immune elicitor, the CP106 coat protein of PVX virus. No Rx1-DNA interaction was observed in the presence of a PVX virus coat protein variant that is incapable of Rx1 activation, nor was it observed when immune signaling was activated via another NLR protein. This finding raises the exciting possibility that a direct NLR-DNA interaction might be a conserved signaling function of activated NLR proteins. To test this hypothesis, we set out to establish whether other NLR proteins are also able to interact with DNA in an activation-dependent fashion. Demonstration of a direct DNA interaction by an alternative NLR provides support that this phenomenon is of broad biological relevance. Further, these experiments will allow an appraisal of the mode of interaction and could reveal NLR-specific differences in nucleic acid interactions.

The I-2 (immunity to race 2) NLR protein from tomato, conferring resistance to tomato wilt disease caused by the fungus *Fusarium oxysporum* f.sp. *lycopersici*, is one of the best studied NLR proteins (16, 38). I-2 is a member of the CC-NB-LRR class of plant NLR proteins that consists of an N-terminal CC domain fused to an NB-LRR domain (39). The CC-NB-ARC domain has been heterologously produced in *Escherichia coli* and shown to bind specifically to adenosine nucleotides and to possess ATPase activity *in vitro* (38). Specific amino acid substitutions in its catalytic site that compromise its ATPase activity result in a mutant I-2 protein that confers an autoimmune phenotype when expressed *in planta* (16). The I-2 protein recognizes the *F. oxysporum* f.sp. *lycopersici* produced Avr2 protein inside the plant nucleus (40), making I-2 a prime candidate NLR protein to have a nuclear signaling activity like Rx1, MLA, RPS4, RPS5-RRS1, SNC1, and N (23–27, 29, 41, 42).

Here we demonstrate that the I-2 NLR protein of tomato is able to bind, bend, and melt duplex DNA *in vitro*. We find important differences between the Rx1-DNA and I-2-DNA interaction; I-2 shows a nucleic acid-binding specificity distinct from Rx1 but similar to the orphan Os02g_25900 NLR of rice. Furthermore, in contrast to Rx1, the I-2 interaction with DNA is coupled to its nucleotide-dependent activation cycle. This directly links DNA interactions with the NLR activation state. We propose that NLR-DNA interactions are a general phenomenon but with NLR-specific differences in the mode of DNA interaction.

## Experimental Procedures

### 

#### 

##### Structural Modeling

Protein fold searches using the Phyre^2^ protein homology/analogy recognition engine, version 2.0 (43) were undertaken using amino acids 175–519 of I-2 and amino acids 197–334 of Os02g_25900 using both normal and intensive modeling modes. Similar structural homology was also detected using the SAM-T08, HMM-based protein structure prediction server (44). Side chain packing and energy minimization was performed using GalaxyRefine (45). The figures were generated using the PyMOL molecular graphics system (46).

##### Protein Expression and Purification

Proteins corresponding to I-2_1–519_^WT^ and I-2_1–519_^K207R^ were generated as described previously (38). The NB subdomain of Os02g_25900 (amino acids 197–334; R1-NB) was generated as previously described (18). Orc1-1 and Orc1-3 of *Sulfolobus solfataricus* were expressed and purified as previously described (47).

##### ATPase Assays

ATPase assays were typically performed at 37 °C for 30 min with 2.3 μm protein in 50 mm 1,3-bis(tris(hydroxymethyl)methylamino) propane, pH 7.5, 10 mm MgCl_2_, and 5 μm ATP. Reactions were spiked with 0.5 μCi of 2,8-^3^H-labeled ATP for quantitation. Reactions were spotted onto a silica thin layer chromatography plate with 1 mm ADP to act as both marker and carrier. The plates were developed in isobutanol:3-methyl-1-butanol:2-ethoxyethanol:ammonia:H_2_O (9:6:18:9:15). Spots were visualized at 256 nm and quantified using an AR-2000 TLC scanner.

##### Electrophoretic Mobility Shift Assays

The oligonucleotides used for quantitative EMSA are derived from a series of oligonucleotides that enables a comparison of relative DNA binding affinity to varying DNA topologies independent of DNA sequence (48). The oligonucleotides sequences were 5′-TGG GTC AAC GTG GGC AAA GAT GTC CTA GCA ATG TAA TCG TCT ATG ACG TT-3′ (SS1; DNA sense-strand), 5′-AAC GTC ATA GAC GAT TAC ATT GCT AGG ACA TCT TTG CCC ACG TTG ACC CA-3′ (SS2; DNA antisense-strand), and 5′-UGG GUC AAC GUG GGC AAA GAU GUC CUA GCA AUG UAA UCG UCU AUG ACG UU-3′ (RNA sense-strand) (47). Oligonucleotides were end-labeled with 10 μCi of [γ-^32^P]ATP using T4 polynucleotide kinase, and unincorporated nucleotides were removed using Micro Bio-Spin columns (Bio-Rad). Protein and 0.15 nm nucleic acids (oligonucleotide 1-ssDNA, annealed oligonucleotide 1 and 2-dsDNA, and ssRNA) were incubated in 20 mm Tris-HCl, pH 8.0, 60 mm NaCl (unless otherwise stated), 2 mm EDTA, 1 mm DTT, 10% (v/v) glycerol, 0.1 mg/ml BSA for 20 min on ice. Quantitative EMSAs were separated on a native 7% (w/v) polyacrylamide gel. Experiments to assess the role of nucleotides on DNA binding used binding reactions and gels supplemented with 10 mm ZnCl_2_ and nucleotide. Polyacrylamide gels were dried and analyzed by autoradiography. EMSAs using unlabeled virion DNA were separated using 0.8% (w/v) Tris acetate-EDTA agarose gels and stained with ethidium bromide. All reported values for *K_d_* represent apparent *K_d_* because of the potential for dissociation of protein-DNA complexes during electrophoresis. Curves were fitted by nonlinear regression in GraphPad Prism 6.0.

##### Fluorescence Anisotropy

Changes in anisotropy were measured using a Carey Eclipse fluorescence spectrophotometer (Agilent Technologies) fitted with polarizing filters (λ_em_ = 520 nm, λ_ex_ = 495 nm, bandwidth = 5 nm, averaging time = 20 s). Anisotropy was determined using a fluorescein end-labeled oligonucleotide of sequence 5′-TGG GTC AAC GTG GGC AAA GA-3′ (SS1s; DNA sense-strand) and an unlabeled oligonucleotide of sequence 5′-TCT TTG CCC ACG TTG ACC CA-3′ (SS2s; DNA antisense-strand) (Eurofins MWG). Oligonucleotides were annealed by heating to 90 °C for 3 min in 10 mm Tris, pH 8.0, 1 mm EDTA before cooling to room temperature. Protein was titrated into 10 nm labeled oligonucleotide in 6.7 mm Tris-HCl, pH 8.0, 3.3 mm sodium acetate, 2 mm EDTA at 25 °C. Anisotropy was calculated using WinFLR software (Agilent Technologies).

##### P_1_ Nuclease Sensitivity

The oligonucleotides for P_1_ nuclease sensitivity were 5′-CTC AAT ACA ATT GTC TCT GTG TAA ATT TCC TAC GTT TCA TCT GAA AAT CTA GCT ATT AGA GCT TGG TTT A-3′ (sense-strand) and 5′-TAA ACC AAG CTC TAA TAG CTA GAT TTT CAG ATG AAA CGT AGG AAA TTT ACA CAG AGA CAA TTG TAT TGA G-3′ (antisense-strand) and represent the C3/mORB dual site sequence at *oriC2* of *S. solfataricus* (49). The sense strand oligonucleotide was end-labeled with 10 μCi of [γ-^32^P]ATP as described above, and sense and antisense oligonucleotides were annealed as required. The reactions were performed in 20-μl volumes containing 20 mm Tris acetate, pH 7.5, 10 mm magnesium acetate, 100 mm NaCl, 0.15 nm oligonucleotide, and 1.5 μm protein. Protein was allowed to bind for 10 min at 37 °C. P_1_ nuclease was added to a final concentration of 0.01–0.1 units μl^−1^ and incubated for a further 20–60 min at 37 °C. The reactions were terminated with 5 μl of 100 mm Tris-HCl, pH 8.0, 2.5% (w/v) SDS, 100 mm EDTA, 10 units μl^−1^ proteinase K. 5 μl of loading buffer (97.5% (v/v) formamide, 10 mm EDTA, 0.3% (w/v), 0.3% bromphenol blue) was added, and the reactions were electrophoresed on a 15% (w/v) polyacrylamide gel containing 8 m urea. Polyacrylamide gels were dried and analyzed by autoradiography. The BSA and ORC control lanes presented in Fig. 4 are identical to those previously published as the I-2 and Rx1 samples have been analyzed side by side (37).

##### Steady-state FRET in Vitro

Synthetic oligonucleotides, unlabeled or end-labeled with fluorescein or tetramethylrhodamine (TAMRA), were purchased from Eurofins MWG. The oligonucleotides used were 5′-TGG GTC AAC GTG GGC AAA GA-3′ (SS1s; DNA sense-strand) and 5′-TCT TTG CCC ACG TTG ACC CA-3′ (SS2s; DNA antisense-strand). Oligonucleotides were annealed by heating to 90 °C for 3 min in 10 mm Tris, pH 8.0, 1 mm EDTA before cooling to room temperature. Steady-state FRET was measured using a PerkinElmer LS 55 fluorescence spectrometer at room temperature in a final volume of 50 μl with 50 nm DNA. Measurements used 1.5 μm protein and were incubated for 10 min at room temperature before scanning. A bandwidth of 5 nm was used for both excitation and emission wavelengths. Donor (fluorescein) was excited at 494 nm, and emission spectra were collected from 490 to 650 nm. Acceptor (TAMRA) was directly excited at 558 nm, and emission spectra were collected from 558 to 650 nm. Spectra were collected for both donor and donor-acceptor-labeled double-stranded DNA, and FRET values were calculated from the increase in acceptor emission using the ratio_A_ method (50). Ratio_A_ was used to calculate energy transfer efficiency (*E*) using the equation *E* = (ϵ*_A_*[558]/ϵ*_D_*[494]) × ratio_A_ − (ϵ*_A_*[494]/ϵ*_A_*[558]) where ϵ*_A_*[λ] and ϵ*_D_*[λ] are the acceptor and donor extinction coefficients provided by the supplier. Donor-acceptor distances (*R*) were calculated using the equation *E* = *R*_0_^6^/(*R*_0_^6^+*R*^6^) and a calculated Förster distance (*R*_0_) of 49.99 Å. The induced bend angle (θ_T_) was calculated using a single-point bend model.

##### Time-resolved FRET in Vitro

Oligonucleotides were as for steady-state FRET *in vitro* and annealed in the same manner. Strands were annealed by heating to 90 °C for 3 min in 10 mm Tris, pH 8.0, 1 mm EDTA before cooling to room temperature. Measurements used 1.5 μm protein with 50 nm DNA in the presence of 60 mm NaCl and were incubated for 10 min at room temperature before analysis. Time-resolved FRET was assessed using the time-correlated, single photon counting technique. The excitation source was a Picoquant pulsed diode laser LDH-P-C-485 (excitation wavelength, 485 nm; 70-ps pulse FWHM at 20 MHz). Fluorescence was detected using an avalanche photodiode (Id Quantique 100-50) linked to a Becker and Hickl SPC 130 time-correlated, single photon counting module. An instrument response function of ∼200 ps was measured from Rayleigh scattered light. Fluorescence decays were collected for both donor and donor-acceptor-labeled double-stranded DNA with or without protein using band pass filter detection of the donor emission and at magic angle polarization.

Data were analyzed by the Grinvald-Steinberg method (51) to obtain the fluorescence lifetime for the donor and acceptor-labeled (τ_DA_) and donor only-labeled (τ_D_) oligonucleotides. The data were fitted to a sum of exponentials using an iterative least squares reconvolution procedure with the optical/electrical excitation profile to produce a biexponential decay containing two lifetimes. This profile was obtained from a slide covered with Silica ludox particles, which provides an instant scatter of the excitation pulse. This data-fitting method provided more accuracy in the determination of shorter lifetimes than calculating a single average lifetime. Efficiencies of energy transfer were calculated from time-resolved FRET according to *E* = 1 − (τ_DA_/τ_D_). Energy transfer efficiencies in the lifetime analysis were higher than those from steady-state experiments as previously observed (52–54). Donor-acceptor distances (*R*) were calculated using the equation *E* = *R*_0_^6^/(*R*_0_^6^+*R*^6^) and a calculated Förster distance (*R*_0_) of 49.99 Å. The total length of the oligonucleotide with linkers and fluorescent dyes, at maximum extension, was calculated as 81.1 Å. The induced bend angle (θ_T_) was calculated using a single-point bend model.

##### Statistical Analysis

*Error bars* represent the standard error of the mean with the number of replicates as indicated in the legend. Statistical comparisons (*p* values) for data that passes a test for normality (D'Agostino and Pearson omnibus normality test and Shapiro-Wilk normality test) were obtained from one-way analysis of variance with the indicated post hoc test. Statistical comparisons (*p* values) for data that do not pass a test for normality were obtained from a Kruskal-Wallis test with post hoc multiple comparisons test. *p* values in statistical comparisons are indicated in the figures with letters and indicate compared data sets as described in the figure legends.

## Results

### 

#### 

##### I-2 Binds Nucleic Acids in Vitro

**FIGURE 1.**
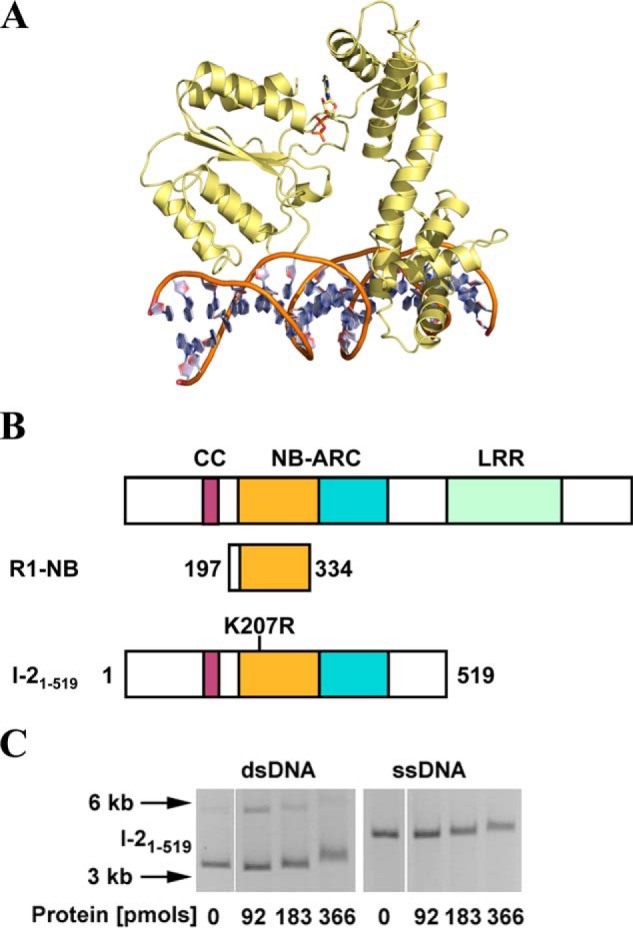
**The I-2 CC-NB-ARC domains bind nucleic acids *in vitro*.** *A*, structural homology model for amino acids 175–519 encompassing the NB-ARC domain of I-2, with associated ADP, bound to DNA. *B*, proteins used in this study. The *top bar* represents a generic CC-NB-LRR type R protein. Conserved domains are *highlighted*. The NB subdomain of the NB-ARC domain containing the P-loop is shown in *orange*, and the tandem ARC subdomains are shown in *cyan*. The *bottom bars* depict the domain composition of the proteins used, with amino acid positions delineating the relevant regions cloned. *C*, EMSA for I-2_1–519_^WT^ using 100 ng of ΦX174 DNA (ssDNA) or ΦX174 RF I DNA (dsDNA). For dsDNA, two bands are visible. The *upper band* represents relaxed circular DNA, whereas the *lower band* represents supercoiled circular DNA. Note that both bands shift in the presence of I-2_1–519_^WT^. Molecular weight markers are indicated with *arrows*.

The NB-ARC domain of Rx1 (amino acids 143–488) has significant homology with the Cdc6/Orc1 proteins of *Pyrobaculum aerophilum* (Protein Data Bank code 1FNN) and of *Aeropyrum pernix* (Protein Data Bank code 2V1U) in complex with DNA (37). We therefore investigated whether there is a similar structural homology in I-2. Amino acids 175–519 of I-2, encompassing the NB-ARC domain, were analyzed using the Phyre^2^ protein fold recognition engine, and the expected close matches with the pro-apoptotic proteins CED-4 (Protein Data Bank code 2A5Y) and Apaf-1 (Protein Data Bank code 1Z6T) were recovered to 100% confidence (55, 56). Like Rx1, high scoring matches (>99% confidence) were also obtained with the Cdc6/Orc1 protein family members. Residues in the NB subdomain and tandem ARC domains were conserved between Cdc6/Orc1 of *A. pernix* and I-2 (29.7% similarity and 9.9% identity). Both the N-terminal NB (amino acids 22–193) and C-terminal ARC domains (amino acids 194–388) in Cdc6/Orc1 contact DNA, inducing deformation of the double helix (47, 49), and the modeled tertiary structure of I-2 closely matched that of Cdc6/Orc1 (Fig. 1*A*). Structural modeling therefore suggests that the NB-ARC domain of I-2 could contact DNA in a manner similar to that of Cdc6/Orc1 and as hypothesized for Rx1. We therefore investigated whether I-2 is also a DNA-binding protein.

A possible direct I-2-DNA interaction was investigated through *in vitro* experiments. EMSA using nucleic acid fragments of >5 kb derived from circular bacteriophage φX174 (57) represents a standard methodology to qualitatively assess interactions between a protein and either ssDNA or dsDNA with identical sequences. EMSAs were performed using recombinant wild type I-2 protein (I-2_1–519_^WT^) encompassing the CC-NB-ARC region but lacking the LRR (Fig. 1*B*). EMSA experiments performed with I-2_1–519_^WT^ showed a direct association with both ssDNA and dsDNA, producing an upward shift in the migration of the nucleic acids similar to that of Rx1 and other unrelated DNA-binding proteins (Fig. 1*C*) (18, 58). I-2 is therefore able to interact with both ssDNA and dsDNA in a sequence-independent manner.

The I-2-DNA interaction was relatively stable because it could be visualized after gel electrophoresis (Fig. 1*C*). Nevertheless, whereas EMSA using circular bacteriophage φX174 DNA is a useful method to qualitatively assess the interaction, it does not enable robust quantification of the affinity of I-2 for nucleic acids. We therefore shifted to EMSAs using small synthetic oligonucleotides to quantify I-2-nucleic acid interactions (59). EMSA with oligonucleotides provides more robust band shifts on EMSA because of their lower molecular mass.

**FIGURE 2.**
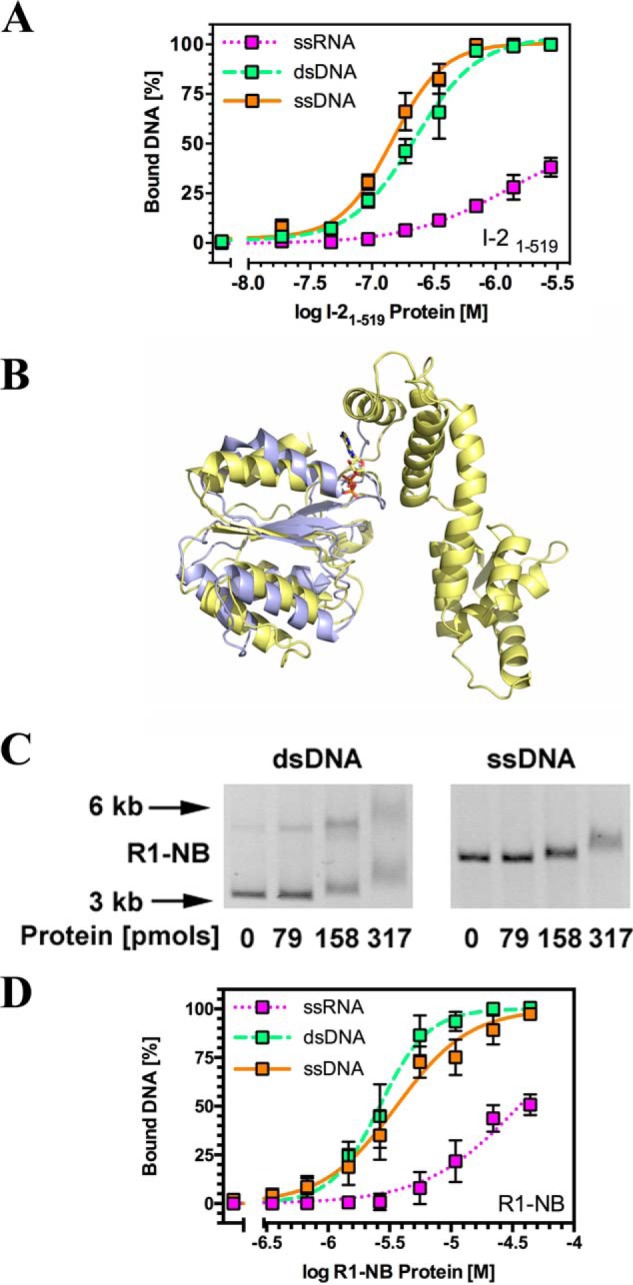
**The I-2 CC-NB-ARC domain and the R1 NB domain bind nucleic acids *in vitro*.** *A*, quantitative EMSA analysis giving affinities of I-2_1–519_^WT^ for synthetic oligonucleotides corresponding to different nucleic acids (*n* = 3–6, ±S.E.). *B*, overlay of a structural homology model for amino acids 197–334 encompassing the NB domain of R1-NB (*blue*) onto the I-2 NB-ARC domain model of Fig. 1*A* (*yellow*). *C*, EMSA for R1-NB using 100 ng of ΦX174 DNA (ssDNA) or ΦX174 RF I DNA (dsDNA). For dsDNA, the *upper band* represents relaxed circular DNA, whereas the *lower band* represents supercoiled circular DNA. Note that both bands shift upon incubation with the R1 NB domain. Molecular weight markers are indicated with *arrows. D*, quantitative EMSA analysis giving affinities of R1 NB for various synthetic oligonucleotides corresponding to different nucleic acids (*n* = 3–10, ±S.E.).

The affinity of I-2_1–519_^WT^ was assessed using ^32^P-labeled synthetic oligonucleotides whose sequences were unrelated to that of bacteriophage φX174 DNA. I-2_1–519_^WT^ showed equivalent affinities for ssDNA and dsDNA but a reduced affinity for ssRNA (Fig. 2*A* and Table 1). The ordering of affinities of I-2_1–519_^WT^ for nucleic acid (ssDNA ≈ dsDNA > ssRNA) is distinct from those of Rx1 (ssDNA > ssRNA > dsDNA), implying that different NLRs can have distinct nucleic acid binding properties *in vitro*. One caveat to this observation is that I-2 is a refolded recombinant protein. Hence, its altered nucleic acid specificity might be a reflection of its refolded status. We therefore investigated the nucleic acid binding specificity of a natively folded NB-ARC subdomain.

**TABLE 1:** **Apparent dissociation constants for recombinant NLR domain interactions with nucleic acids** The error values represent the standard deviation. ND, not determined.

Protein	Method	*K_d_*		
		ssDNA	dsDNA	ssRNA
		μ*m*	μ*m*	μ*m*
I-2_1–519_^WT^	EMSA	0.15 ± 0.01	0.22 ± 0.02	>100
I-2_1–519_^K207R^	EMSA	ND	0.23 ± 0.01	ND
I-2_1–519_^WT^	Anisotropy	ND	0.32 ± 0.05	ND
I-2_1–519_^K207R^	Anisotropy	ND	0.94 ± 0.09	ND
R1-NB	EMSA	3.34 ± 0.54	2.75 ± 0.40	12.45 ± 2.34

We were unable to produce the individual NB or NB-ARC subdomains of I-2 in *E. coli* and therefore shifted to the isolated NB subdomain of an I-2 homolog, the orphan NB-LRR protein Os02g_25900 (R1-NB). R1-NB was selected for analysis because it has been previously demonstrated to be readily expressed as a natively folded protein in *E. coli* (18). R1-NB therefore avoids the criticism that its activity might be affected by the *in vitro* refolding procedure that could result in a subfraction of potentially mis- or unfolded protein. Furthermore, structural modeling demonstrated a close structural similarity between the NB domains of R1-NB and that of I-2 (Fig. 2*B*). Like I-2_1–519_^WT^, R1-NB bound both circular bacteriophage φX174 ssDNA and dsDNA in a sequence-independent fashion (Fig. 2*C*). R1-NB interacted with a variety of oligonucleotides with an ordering of affinities for nucleic acids of ssDNA ≈ dsDNA > ssRNA (Fig. 2*D* and Table 1). The ranking for R1-NB is similar to that observed for I-2_1–519_^WT^ with a clear preference for binding DNA over RNA. We hence conclude that the nucleic acid affinities for I-2 are likely a reflection of its genuine biochemistry and not an artifact of refolding the recombinant protein.

**FIGURE 3.**
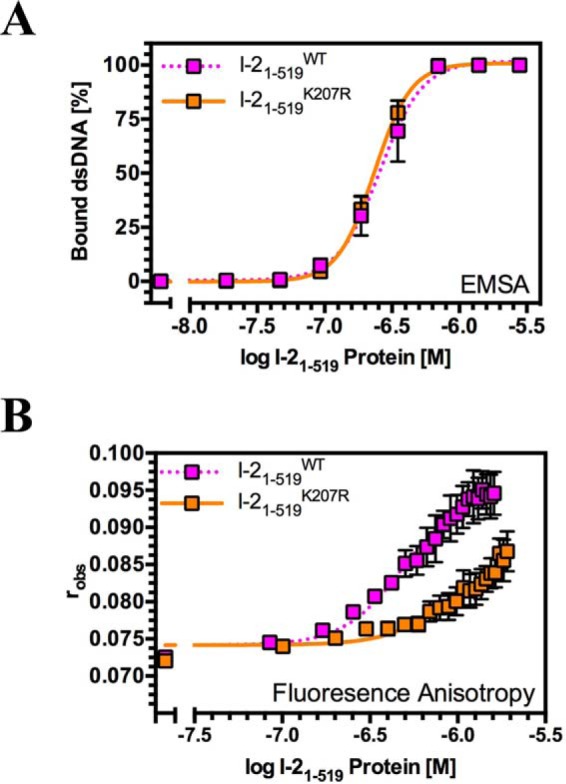
**The I-2 K207R mutant shows an altered mode of interaction with dsDNA in comparison with wild type I-2.** *A*, quantitative EMSA analysis giving comparative affinities of I-2_1–519_^WT^ and I-2_1–519_^K207R^ for synthetic dsDNA oligonucleotides (*n* = 3, ±S.E.). *B*, fluorescence anisotropy analysis giving distinct affinities of I-2_1–519_^WT^ and I-2_1–519_^K207R^ for dsDNA (*n* = 3, ±S.E.) suggestive for a different topology of the protein-DNA complex.

The P-loop mutation K207R of I-2 (I-2_1–519_^K207R^) reduces the *K_m_* for ATP *in vitro* and confers a loss of function phenotype *in vivo* (38). We therefore set out to test whether the I-2 K207R mutant shows a similar distinct pattern of interaction when compared with the wild type protein. We first compared the affinity of I-2_1–519_^WT^ and I-2_1–519_^K207R^ for dsDNA. Using an EMSA assay, the affinities of I-2_1–519_^WT^ and I-2_1–519_^K207R^ for dsDNA were largely indistinguishable (Fig. 3*A*). However, when the affinity of I-2_1–519_^WT^ and I-2_1–519_^K207R^ was compared using fluorescence anisotropy, the affinity of I-2_1–519_^WT^ for dsDNA was significantly greater than that of I-2_1–519_^K207R^ (Fig. 3*B* and Table 1). Affinities measured by anisotropy depend not only upon the mass of a protein-DNA complex at a given molar ratio but also on its globular structure. EMSA therefore suggests that the binding constants of I-2_1–519_^WT^ and I-2_1–519_^K207R^ are very similar, but anisotropy reveals that the overall shape of the protein complex on DNA might be different. Together, these data indicate that, like Rx1, I-2 and R1 interact with nucleic acids but that the affinities for different nucleic acids vary for the different NLRs. Further, the structure of the NLR-DNA complex formed in I-2 depends on the presence of an intact P-loop.

##### I-2 Deforms DNA

**FIGURE 4.**
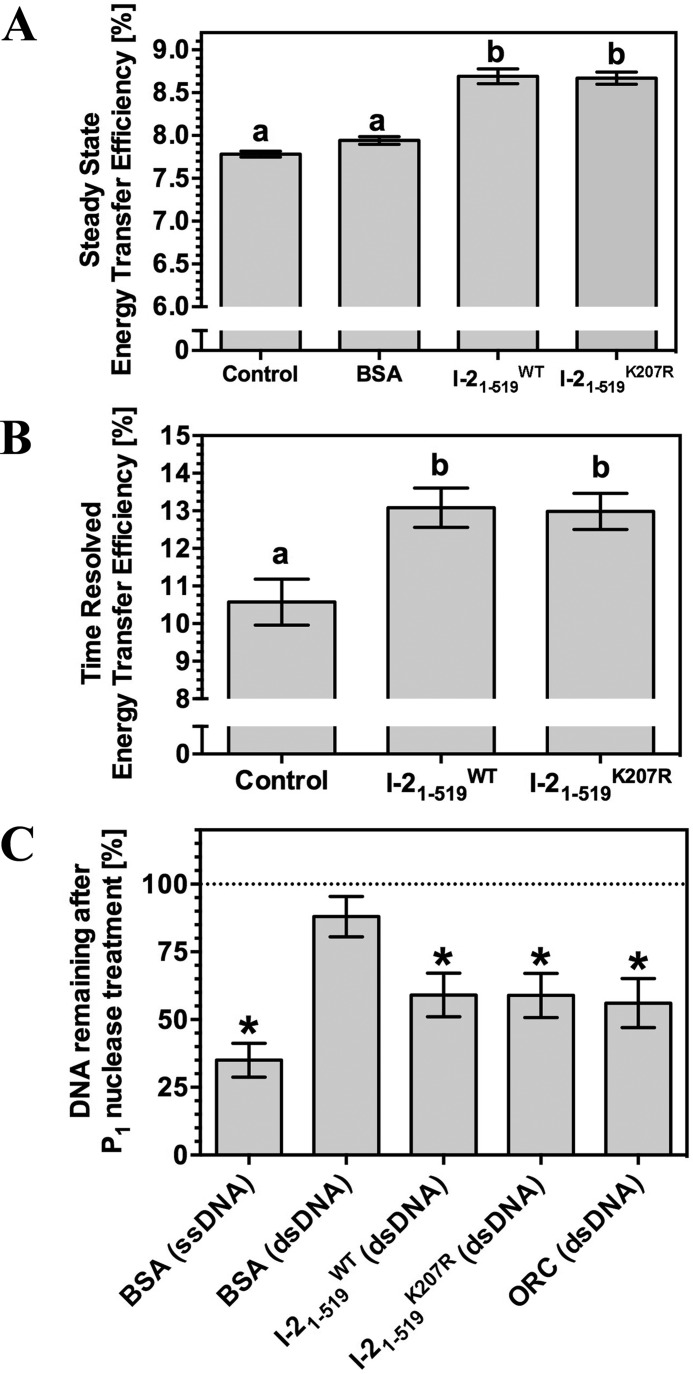
**I-2_1–519_ distorts and melts dsDNA.** *A*, energy transfer efficiency in steady-state FRET in response to I-2_1–519_ binding (*n* = 3, ±S.E.). The control is BSA. *B*, energy transfer efficiency in time resolved FRET in response to I-2_1–519_ binding. *C*, P_1_ nuclease sensitivity of ssDNA and dsDNA following incubation with BSA (negative control), I-2_1–519_, or ORC (positive control). *, *p* < 0.01 compared with control by one-way analysis of variance with post hoc Dunnett test.

The Cdc6/Orc1 family proteins substantially deform origin DNA by bending it with angles of 35 and 20°, respectively, thereby inducing localized melting of the double helix (47, 49, 60). The Rx1 protein is also able to bend dsDNA at an angle of 42° (37). We therefore investigated whether I-2 is able to deform dsDNA in a similar fashion. We examined DNA distortion by steady-state FRET using two-sided end-labeled dsDNA (61). DNA distortion was evident by an increase in FRET acceptor emission in the presence of I-2_1–519_^WT^ or I-2_1–519_^K207R^ when compared with the mock (no protein) or to a control protein (BSA) (Fig. 4*A*). Energy transfer efficiency correlates with the distance between fluorophores and was found to increase in the presence of I-2_1–519_^WT^ or I-2_1–519_^K207R^. The change in efficiency can be used to calculate their distances and revealed that I-2_1–519_^WT^ and I-2_1–519_^K207R^ induced bend angles (θ_T_) of 21.8–23.9 and 22.1–23.1°, respectively (values represent a range of ±S.E. from the mean). We next measured the fluorescence lifetime, which represents an intrinsic property of the fluorophore that is independent of concentration, photobleaching, and light scattering, as an alternative method to measure DNA distortion through a FRET process. The decrease in fluorescent donor lifetime can only be rationalized because of increased energy transfer from donor to the acceptor. Together with the data from steady-state analysis, these data both show increased energy transfer efficiency from donor to acceptor in the presence of I-2_1–519_^WT^ or I-2_1–519_^K207R^ (Fig. 4*B*). The recorded fluorescence lifetimes correspond to θ_T_ values for I-2_1–519_^WT^ and I-2_1–519_^K207R^ of 29.7–36.7 and 29.6–35.1°, respectively (values represent a range of ±S.E. from the mean). In conclusion, I-2_1–519_ distorts DNA like Cdc6/Orc1 and R1. However, the bend angle induced by I-2, as measured by two independent methods, is substantially lower than that induced by Rx1, indicating a similar yet distinct mode of action.

The Orc1 protein of *A. pernix* and Rx1 also induces local DNA distortion upon binding. The P_1_ nuclease can be used to detect distortion specific ssDNA in the presence of the bending proteins (37, 60, 62). We therefore examined the sensitivity of dsDNA oligonucleotides to the ssDNA-specific P_1_ nuclease in the presence of I-2_1–519_. As expected, ssDNA was significantly degraded (positive control), whereas dsDNA in the presence of BSA (negative control) was largely resistant to P_1_ nuclease activity (Fig. 4*C*). dsDNA was more sensitive to P_1_ nuclease in the presence of either I-2_1–519_^WT^or the mutant I-2_1–519_^K207R^, showing that both proteins can melt dsDNA. The P_1_ sensitivity of dsDNA in the presence of Orc1-1/Orc1-3 was indistinguishable from that of dsDNA in the presence of I-2_1–519_, supporting the interpretation that I-2, similar to Rx1, can cause local dsDNA melting (Fig. 4*C*). In conclusion, I-2 is able to bend DNA and provoke localized DNA melting. Because the pattern of DNA distortion induced by I-2 is indistinguishable between I-2_1–519_^WT^and the mutant I-2_1–519_^K207R^, the difference in the nature of the protein-DNA complex is not at the level of melting the DNA.

##### The I-2-DNA Interaction Is Coupled to the Nucleotide Binding State and Activity of the P-loop

**FIGURE 5.**
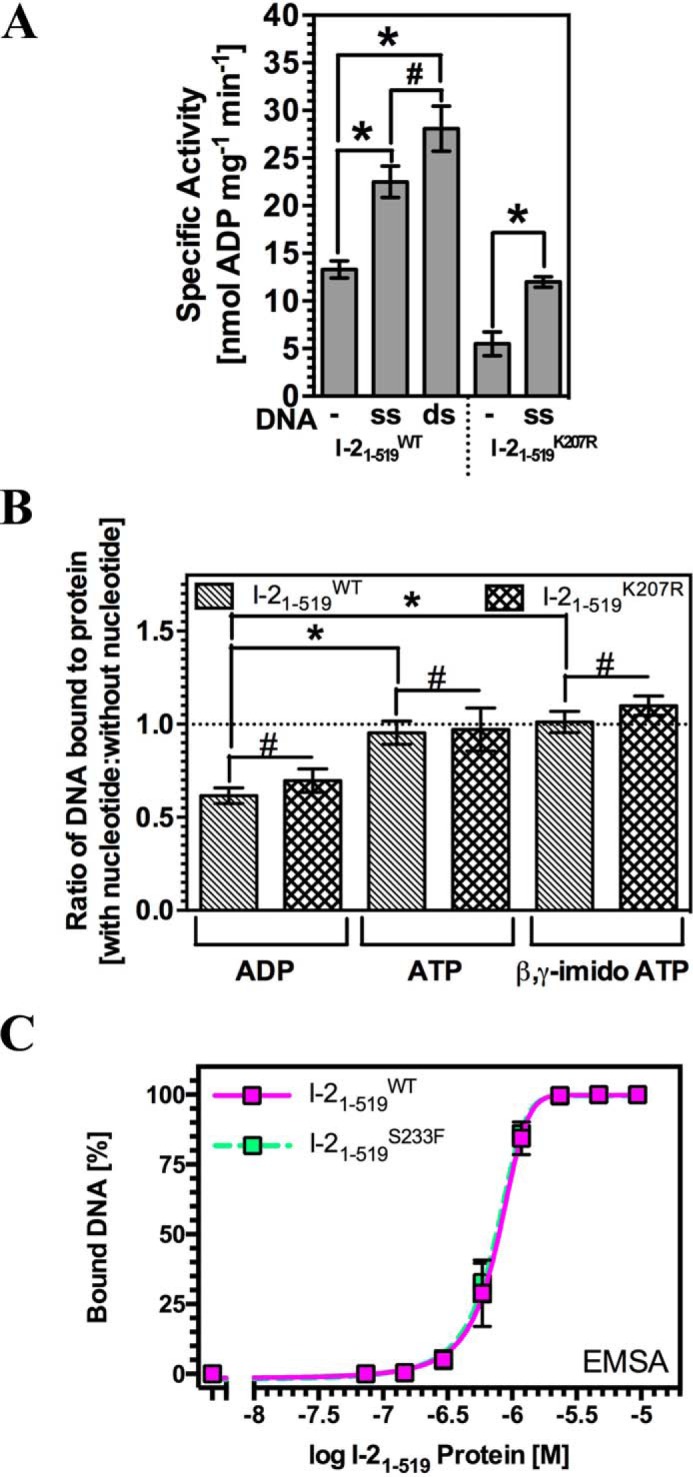
**DNA binding is coupled to the nucleotide binding state and ATPase activity of I-2_1–519_.** *A*, the influence of 2 μm DNA on the intrinsic ATPase activity of I-2_1–519_^WT^ and I-2_1–519_^K207R^ (*, *p* < 0.05; #, *p* > 0.05; *n* = 4, ±S.E.). The source DNA is identical to that used for EMSA in Fig. 2*A. B*, double-stranded DNA binding by I-2_1–519_^WT^ and I-2_1–519_^K207R^ assessed by EMSA plotted as a ratio of binding in the presence of 5 μm nucleotide (ADP, ATP, or β,γ-imido ATP) compared with no nucleotide (*, *p* < 0.05; #, *p* > 0.05; *n* = 4–6, ±S.E.). *C*, quantitative EMSA analysis giving comparative affinities of I-2_1–519_^WT^ and I-2_1–519_^S233F^ for synthetic dsDNA oligonucleotides (*n* = 3, ±S.E.).

The findings on DNA interaction and distortion seem to indicate that the difference between I-2_1–519_^WT^ and the mutant I-2_1–519_^K207R^ is related to the conformational state of the protein (Fig. 3*B*) and not DNA melting (Fig. 4*C*). We therefore sought independent evidence to understand the relationship between the nucleotide occupancy and ATPase activity of the P-loop in I-2 (compromised in I-2_1–519_^K207R^) and DNA binding. In the “switch” model for plant NLR activation, binding of ATP to the NB-ARC domain establishes the on state, whereas hydrolysis of ATP to ADP restores the off state (13). An intact P-loop is essential for nucleotide binding, and mutations in this motif typically result in loss of function alleles (13). We therefore investigated the relationship between P-loop-dependent ATPase activity and DNA binding. The CC-NB-ARC domain of I-2 has a very low intrinsic ATPase activity *in vitro*. Interestingly, the ATPase activity of I-2_1–519_^WT^ was increased 2-fold in the presence of DNA (Fig. 5*A*). Although I-2_1–519_^K207R^ has a reduced ATPase activity, reflecting its lowered *K_m_* for ATP, its enzymatic activity was also stimulated 2-fold by DNA.

Next, we tested the reciprocal relationship and examined whether nucleotides influence DNA binding by I-2_1–519_. To be able to monitor either increased or reduced binding capacity, an I-2_1–519_ protein concentration was used that gives ∼50% of maximal DNA binding. The ratio of I-2_1–519_^WT^ bound to DNA in the presence of 5 μm ATP (giving about 70% nucleotide occupancy for I-2_1–519_^WT^ (16)) compared with DNA-bound I-2_1–519_^WT^ in the absence of nucleotides was approximately unity (Fig. 5*B*). In contrast, the ratio of I-2_1–519_^WT^ bound to DNA in the presence of 5 μm ADP was ∼0.65 as compared with that in the presence of ATP or in the absence of nucleotides, demonstrating a reduced binding of the I-2-ADP complex. Binding of I-2_1–519_^WT^ to DNA in the presence of the nonhydrolyzable ATP analog, adenosine 5′-(β,γ-imido)triphosphate, was indistinguishable from DNA binding in the presence of ATP, ruling out that the enhanced binding, compared with that measured in the presence of ADP is due to hydrolysis of ATP. The influence of nucleotides on I-2_1–519_^K207R^ DNA binding was the same as that for I-2_1–519_^WT^. Because K207R is a *K_m_* mutation that likely effects coordination of the ATP β-PO_4_^2−^, it is not surprising that this mutant shows a similar response to the nucleotides. Because the ATP and “no nucleotide” bound states appear equivalent in this assay, it was also to be expected that this mutant behaved similarly to the wild type, despite its lowered *K_m_* for ATP.

To provide further evidence that the difference between the DNA binding ability of the ATP and ADP-bound states is not due to changes in nucleotide handling, we investigated an additional I-2 mutant. The S233F mutant is autoactive *in vivo* because of its reduced ATP hydrolysis rate, whereas it has an affinity for ATP equivalent to wild type (16). Binding of I-2_1–519_^S233F^ to dsDNA was indistinguishable from I-2_1–519_^WT^ (Fig. 5*C*). We found that for I-2_1–519_^S233F^, similar to wild type I-2, DNA binding in the presence of 5 μm ADP was only 61 ± 7% (S.D.) of that in the presence of 5 μm ATP. This observation further supports our hypothesis that DNA binding by I-2 is enhanced by ATP relative to ADP and that this change cannot be attributed to differences in nucleotide handling by the different proteins.

Together these results indicate that the intrinsic ATPase activity of I-2 is enhanced in the presence of DNA. This converts the ATP-bound state into the ADP-bound conformation with a consequent increased likelihood for release from DNA.

## Discussion

Immune responses induced by NLR immune receptors are broadly conserved (63), suggesting a common signaling network that might be activated via a conserved mechanism. Existing *in vitro*, *in vivo*, and bioinformatics data identify the NB-ARC domain as the most conserved domain in NLR proteins. The NB-ARC domain functions as a molecular switch regulating NLR activity. Its conservation makes the NB-ARC domain also a prime candidate to be involved in activating the downstream signaling process. The NB-ARC domain of the potato Rx1 NLR protein was shown to possess intrinsic DNA binding activity that is activated *in situ* only upon triggering the cellular immune response by admission of the pathogen-derived elicitor (37). Here we demonstrate that both R1 of rice and I-2 of tomato are also able to interact with nucleic acids *in vitro*. This study therefore establishes nucleic acid interactions as central to the biochemistry for at least a subset of NLR proteins. The observed specificity for ssDNA, dsDNA, and RNA for these proteins revealed subtle differences in their abilities to interact with nucleic acids. The functional relevance of these differences awaits future elucidation.

I-2 shares many biochemical properties with Rx1 and the Cdc6/Orc1 family of DNA-binding proteins. I-2 was observed to bind both ssDNA and dsDNA similar to ORC of *Saccharomyces cerevisiae* and Rx1 (37, 64). Based upon the Cdc6/Orc1 homology with NLR proteins, and the DNA binding characteristics of the isolated R1 NB domain (Fig. 2*B*), it is most likely that this domain is also the site of DNA binding in the I-2_1–519_ protein, although a role for the CC domain cannot be formally excluded. Unfortunately it was not possible to generate truncated I-2 protein lacking the CC domain to test this hypothesis. Eukaryotic ORCs and Rx1 lack DNA sequence specificity *in vitro*, and this property is also shared with I-2. Superficially, therefore, I-2 biochemistry and ability to interact with nucleotides appears similar to Rx1. Similar to Rx1, the I-2 protein used for analysis is lacking its LRR domain region. The absence of a domain resembling the LRR in Cdc6/Orc1 proteins validates the use of this truncated protein in assessing DNA binding in NLR proteins. However, we cannot exclude the possibility that the LRR might influence the affinity of the NB-ARC region for DNA through constraints placed on relative domain orientations. These experiments are an important future target that first require the synthesis of significant amounts of full-length NLR protein, currently a considerable technical challenge.

A number of crucial differences, however, between the biochemistry of Rx1 and I-2 are observed. First, Rx1 and I-2 show divergent specificity for different nucleic acid species. Rx1 has a higher affinity for single-stranded nucleic acids (ssDNA and RNA) over double-stranded nucleic acids, whereas I-2 has a higher affinity for dsDNA over RNA. The significance of this for the respective NLRs will await future functional characterization. Both Rx1 and I-2 distort dsDNA but to differing extents. The bend angle introduced into DNA by I-2 (22–33°) is substantially less than that introduced by Rx1 (42°) but of a similar magnitude as that introduced by Orc1-1/Orc1-3 of *S. solfataricus* (20°) and ORC1 of *A. pernix* (35°) (37, 47, 49). The most noticeable difference between Rx1 and I-2, however, is that I-2 allows us to make a coupling between its P-loop—required for the switch function—and DNA binding. In contrast to Rx1, I-2 has a measurable ATPase activity, which we found to be stimulated by DNA (Fig. 5*A*). Furthermore, (nonhydrolyzable) ATP was demonstrated to promote I-2 binding to DNA relative to the ADP-bound state. This observed property is consistent with the switch hypothesis for NB-ARC domain activation stating that the “open” ATP-bound state is the active state triggering immune signaling, whereas the “closed” ADP-bound conformation corresponds to the autoinhibited state (8). Increased DNA binding in the presence of ATP and a nonhydrolyzable ATP analog, when compared with ADP, suggests that DNA binding is stimulated in response to NLR activation upon effector recognition. That ATP-bound and non-nucleotide-bound I-2 show similar binding to DNA suggests that the structure of the empty protein resembles that of the activated state and differs from that the “closed” ADP bound confirmation. Incubation with DNA stimulates the ATPase activity of I-2, suggesting that DNA binding is a self-limiting process, and it is tempting to speculate that DNA binding and release is a cyclic process triggered by the presence of the effector. A similar cyclic mechanism, allowing multiple rounds of elicitor recognition providing a means for signal amplification, has been proposed before for Rx and was based on interaction studies of the CC-NB-ARC and LRR domains (65). Further study is required to assess the significance and generality of these findings for other NLRs.

Additional evidence linking coupling of the activation state and switch function of the NB-ARC to DNA binding comes from the analysis of an I-2 P-loop mutant. Wild type I-2 and a variant with a defective P-loop have a similar affinity for double-stranded DNA as assessed by EMSA. However, measurements using fluorescence anisotropy reveal a reduced affinity for the P-loop mutant. These data suggest an overall different shape for the protein-DNA complex for the wild type and P-loop mutant proteins. This difference is not due to alterations in conformation of the bound DNA because the observed pattern of DNA distortion, measured through DNA bending and melting, is identical in both cases. It is reasonable to assume, therefore, that the difference in topology is attributed to the protein. In support of this hypothesis, equivalent mutations in the NB domain of Cdc6 have been shown to affect its ability to interact with other proteins and form complexes at dsDNA (66, 67). It is formally possible that anisotropy, but not EMSA, detects an I-2 complex that forms for the wild type, but not the mutant protein. Such a complex could result in a higher observed anisotropy. These observations provide insight into the molecular mechanism for how P-loop mutations, commonly used to investigate the role of NLR switch function in immunity, might exert their activity. NLR variants with P-loop modifications, such as the I-2 K207R mutant, showing a decreased *K_m_* [ATP] are defective in immune activation. At cellular [ATP], this defect may not necessarily contribute to decreased ATP binding but may be a reflection of an altered conformational state that could influence trafficking (29) or its activity at DNA (66, 67). Unfortunately, chimeric fusions of I-2 with GFP are nonfunctional, so we cannot use microscopic methods to investigate the effect P-loop mutants have on I-2-mediated DNA binding *in planta* in relation to immune signaling (37).

In summary, we have identified a DNA binding and distorting activity in the I-2 protein *in vitro*. This establishes nucleic acid binding as a conserved biochemical feature for at least a subset of plant NLR proteins (I-2, R1, Rx1, and PSiP). A further conserved feature of these NLR proteins in DNA manipulation is through bending and melting of the double helix. We further establish that individual NLR proteins distinctively differ in the interactions with various nucleotide topologies (ssDNA, dsDNA and RNA) that might arise from their functional diversity. DNA binding in I-2 is directly linked to the switch function of its central NB-ARC domain, thus directly linking activity at DNA to known biochemical requirements for NLR activation.

## Author Contributions

S. F., P. D. T., C. H. D., and W. H. G. performed the experiments. S. F., P. D. T., C. H. D., W. H. G., G. J. S., L. O. P., F. L. W. T., and M. J. C. analyzed the data. G. J. S., L. O. P., F. L. W. T., and M. J. C. conceived the experiments. M. J. C. and F. L. W. T. conceived the overall project and wrote the manuscript. All authors reviewed the results and approved the final version of the manuscript.

## Footnotes

3 The abbreviations used are:

NLR: nucleotide-binding leucine-rich repeat
CC: coiled-coil
NB: nucleotide-binding
NB-ARC: nucleotide-binding, Apaf-1, R-proteins, and CED-4
LRR: leucine-rich repeat
ssDNA: single-stranded DNA.
